# Downregulation of female *doublesex* expression by oral-mediated RNA interference reduces number and fitness of *Anopheles gambiae* adult females

**DOI:** 10.1186/s13071-019-3437-4

**Published:** 2019-04-15

**Authors:** Mabel L. Taracena, Catherine M. Hunt, Mark Q. Benedict, Pamela M. Pennington, Ellen M. Dotson

**Affiliations:** 1 Centers for Disease Control and Prevention (CDC), 1600 Clifton Road, NE, Atlanta, GA 30329-4027 MS G49, USA; 2 Centro de Estudios en Biotecnologia, Universidad del Valle de Guatemala, 18 Avenida 11-95, 01015 Guatemala City, Guatemala

**Keywords:** *Anopheles gambiae*, *doublesex*, RNAi, Gene silencing, Mosquito rearing, Female-specific, Sex determination

## Abstract

**Background:**

Mosquito-borne diseases affect millions worldwide, with malaria alone killing over 400 thousand people per year and affecting hundreds of millions. To date, the best strategy to prevent the disease remains insecticide-based mosquito control. However, insecticide resistance as well as economic and social factors reduce the effectiveness of the current methodologies. Alternative control technologies are in development, including genetic control such as the sterile insect technique (SIT). The SIT is a pivotal tool in integrated agricultural pest management and could be used to improve malaria vector control. To apply the SIT and most other newer technologies against disease transmitting mosquitoes, it is essential that releases are composed of males with minimal female contamination. The removal of females is an essential requirement because released females can themselves contribute towards nuisance biting and disease transmission. Thus, females need to be eliminated from the cohorts prior to release. Manual separation of *Anopheles gambiae* pupae or adult mosquitoes based on morphology is time consuming, is not feasible on a large scale and has limited the implementation of the SIT technique. The *doublesex* (*dsx*) gene is one of the effector switches of sex determination in the process of sex differentiation in insects. Both males and females have specific splicing variants that are expressed across the different life stages. Using RNA interference (RNAi) to reduce expression of the female specific (*dsxF*) variant of this gene has proven to have detrimental effects to the females in other mosquito species, such as *Aedes aegypti*. We tested oral RNAi on *dsx* (*AgdsxF*) in *An. gambiae.*

**Methods:**

We studied the expression pattern of the *dsx* gene in the *An. gambiae* G3 strain. We knocked down *AgdsxF* expression in larvae through oral delivery of double stranded RNA (dsRNA) produced by bacteria and observed its effects in adults.

**Results:**

Our results show that feeding of *AgdsxF* dsRNA can effectively reduce (> 66%) the mRNA of female *dsx* transcript and that there is a concomitant reduction in the number of female larvae that achieve adulthood. Control groups produced 52% (± 3.9% SE) of adult males and 48% (± 4.0% SE) females, while *AgdsxF* dsRNA treated groups had 72.1% (± 4.0% SE) males *vs* 27.8% females (± 3.3% SE). In addition, the female adults produce fewer progeny, 37.1% (± 8.2% SE) less than the controls. The knockdown was sex-specific and had no impact on total numbers of viable male adults, in the male *dsx* transcripts or male fitness parameters such as longevity or body size.

**Conclusions:**

These findings indicate that RNAi could be used to improve novel mosquito control strategies that require efficient sex separation and male-only release of *An. gambiae* by targeting sex determination genes such as *AgdsxF.* The advantages of using RNAi in a controlled setting for mosquito rearing are numerous, as the dose and time of exposure are controlled, and the possibility of off-target effects and the waste of female production would be significantly reduced.

**Electronic supplementary material:**

The online version of this article (10.1186/s13071-019-3437-4) contains supplementary material, which is available to authorized users.

## Background

Despite recent progress in malaria control, the disease remains a global threat. Nearly half of the world’s population is at risk and the disease kills more than 400 thousand people a year [1]. *Anopheles gambiae* (*s.l*.) is the main malaria vector in sub-Saharan Africa, the region with the highest mortality rates, due to the combination of this mosquito’s high anthropophily and its susceptibility to *Plasmodium falciparum* infection [2–4]. Only female mosquitoes transmit the disease, as they ingest the blood meal that is necessary for the completion of their reproductive cycle [5]. Thus, female sex dimorphisms in *An. gambiae* include sex-specific morphological and physiological adaptations for host-seeking, blood-feeding and digestion [6].

Mosquito control to prevent malaria transmission is currently being undermined by the rapid spread of resistance to common insecticide classes [7–9]. Alternative tools and innovative strategies are needed to improve integrated management programmes [10], which rely on complementary strategies to minimize resistance. The sterile insect technique (SIT) is a system based on the mass rearing and release of adult males sterilized using gamma or X-rays to compete with wild males for matings with wild females [11–13]. Because *An. gambiae* females generally are monogamous, after mating with a sterile male, most females would only produce non-viable eggs. As a result, the mosquito population would be significantly reduced after a few generations if sufficient mating with sterile males occurred [14]. The SIT is, however, a self-limiting strategy that depends on the continuous and periodic release of sterile male mosquitoes. The technical difficulties in separating males from females in the mass production required for SIT, as well as the fitness costs of sterilization to males, have limited the implementation of this technique [12–15]. Importantly, the contamination with females should be avoided, because, even if they are sterile, they still may be able to transmit the disease. Furthermore, sex separation techniques are also needed for other methods currently under development, such as the release of *Wolbachia* infected incompatible males as a means of population reduction or the release of genetically modified mosquitoes [16].

The molecular mechanisms responsible for sexual dimorphism in insects include sex-specific gene splicing [17]. The *doublesex*/mab-3 related (Dmrt) family of transcription factors is involved in sex-specific differentiation, working as tissue-specific developmental regulators that integrate information about sex, position and time to guide specific cell types toward male or female differentiation [18–20]. The *doublesex* (*dsx*) gene appears to be conserved as a switch at the bottom of the cascade, and in many insects the sex-specific splicing of the gene is directed by the Transformer protein (TRA) with Transformer-2 (TRA2), which form a TRA/TRA2 complex [21]. The downstream targets of insect *dsx* are not well elucidated, but at least 50 optimal binding sites and genes have been identified for *Drosophila melanogaster doublesex* (*Dmdsx*) [22, 23].

In *An. gambiae*, the sex-specific morphological, physiological and behavioral traits are determined by the differential expression (sex-bias) of genes that are present in both males and females [6]. There is evidence that this differential expression of genes could be regulated by the *An. gambiae dsx* gene (*Agdsx*), with specific splicing for each sex [24, 25], regulating the genes involved in the adaptations to facilitate hematophagy, reproduction, olfaction and immune responses to pathogen challenge [21, 26]. In other mosquito species, such as *Aedes* spp. and *Culex* spp., female-specific *dsx* silencing has had detrimental effects on female development and more recently CRISPR-Cas9 knock-out of the same gene in *An. gambiae* has been shown to masculinize or completely sterilize the females [27–30].

RNA interference (RNAi) is a natural eukaryotic mechanism to silence genes in a post-transcriptional step, without permanent modifications to the genome. The use of exogenous dsRNA allows targeting a specific gene to suppress its expression by triggering the degradation of its complementary mRNA in the cell [31–34]. Previous work by Whyard et al. [29] has successfully used RNAi in larvae to silence genes involved in sex differentiation in *Ae. aegypti*, including *dsx*. In *An. gambiae*, Zhang et al. [35] have used chitosan nanoparticles to deliver dsRNA for chitin synthase genes, but to our knowledge there is no report of *dsx* silencing by RNAi in this species. To study the viability of a knockdown of *dsx* in *An. gambiae* using a system of RNAi by oral delivery, we fed young larvae with dsRNA for *AgdsxF*, to silence the *AgdsxF* and reduce the total number of female individuals in the laboratory setting. This approach can be complemented and modified to eliminate females from the male production without the need for manual sex sorting. Targets such as *AgdsxF* are especially appealing for this technique due to the sequence specificity that the sex-specific splicing provides. This could make the methodology species- and target-specific and would minimize the possibility of off-target effects.

## Methods

### Animal rearing and sexing

Laboratory-reared mosquitoes, *An. gambiae* G3 strain [MRA-112, BioDefense and Emerging Infections (BEI) Malaria Research and Reference Reagent Resource Center (MR4) vector activity, Centers for Disease Control and Prevention (CDC), Atlanta, Georgia, USA] were used for all experiments. Insectary conditions were 27 °C and a relative humidity of 80% with a photoperiod of 12:12 light: dark photocycle with a 30-min dawn and dusk period. Adults were fed *ad libitum* on 10% sugar solution with 0.2% methylparaben dissolved in sterile water. Larvae were fed on Damiens diet [tuna meal, liver powder, and Vanderzant vitamin mix (BioServ, NJ, USA)] in a 2:2:1 ratio; 2% w/v slurry using 10 g with 500 ml water) [36] unless specified otherwise.

Laboratory stocks of other mosquito species used were obtained from the Malaria Research and Reference Reagent Resource Center (MR4). Sex was determined in the pupal stage by observation of the pupal terminalia.

### *Doublesex* sequence selection

GeneBank KM978938 sequence was used to design primers specific for the female mRNA splice form. After confirmation of sequence specificity and expression verification (Additional file 1), primers dsx1586 and dsx1589 were selected for the long dsRNA construct (Table 1). PCR amplification was performed using the AccuStart II PCR Supermix (Quantabio, MA, USA) and 10 µM primers, using the Tm’s specific for them. PCR products were visualized in 1.5% agarose gels stained with ethidium bromide and the amplicon was purified for cycle sequencing using a MultiScreen^®^HTS Vacuum Manifold (Millipore, MA, USA). DNA cycle sequencing reactions were performed for both strands with BigDye 3.1 sequencing kit (Thermo Fisher Scientific, Waltham, MA, USA), following manufacturer standard protocols, and sequenced in an ABI3130 (Applied Biosystems, Foster City, CA, USA) automated capillary sequencer. Sequences were manually assembled and aligned with SeqmanPro (DNASTAR Inc, Madison, WI, USA) and BioEdit 7.2.0. [37] After identity confirmation, T7 tailed primers were used to amplify the product with a T7 tail (either forward or reverse) to clone it into a pGEM-T-easy vector (Promega, WI, USA) according to the manufacturer’s instructions.

**Table 1:** Sequences of PCR primers

Gene	Vector base ID	Primer name	Primer sequence 5′–3′	Efficiency (%)
*Rps7*	AGAP010592	S7qf1	AGAACCAGCAGACCACCATC	115.02
		S7qr1	GCTGCAAACTTCGGCTATTC	
*Actin5C*	AGAP000651	ACT-2f	TACAACTCGATCATGAAGTGCGA	110.67
		ACT-3r	CCCGGGTACATGGTGGTACCGCCGGA	
*Elongation Factor*	AGAP005128	EFf1	GGCAAGAGGCATAACGATCAATGCG	112.60
		EFr1	GTCCATCTGCGACGCTCCGG	
*Female doublesex*	AGAP004050	newDSX-f	AGAGGGCGGGGAAATTCTAGT	111.19
		newDSX-r	GGGCTTGTGGCAGTACGAATA	
*Female doublesex*	AGAP004050	dsRNA_dsx-f2 (dsx1586)	CAAGCGGTGGTCAACGAATA	na
		dsRNA_dsx-r3 (dsx1589)	GCCCACTCCTTAAACACTACTT	
*Female doublesex*	AGAP004050	T7dsRNA_dsx-f2	TAATACGACTCACTATAGGGCAAGCGGTGGTCAACGAATA	na
		T7dsRNA_dsx-r3	TAATACGACTCACTATAGGGGCCCACTCCTTAAACACTACTT	

### RNAi experiments

Due to the sex-specific nature of the *dsx* exons, we selected a female-specific 260 bp fragment within the *dsxF* mRNA sequence and added a T7 sequence to the 3′ end and ligated it to a pGEM-T-easy plasmid to obtain a product flanked by two T7’s for dsRNA production. *Escherichia coli* HT115 (DE3) was transformed with T7 plasmids as described by Timmons [31] to express the selected dsRNA for the *dsx* gene. An *E. coli* HT115 (DE3) previously transformed [38] to produce an unrelated dsRNA for the *Aintegumenta* gene from *Arabidopsis thaliana* was used as control in all the feeding experiments. For each feeding, an overnight culture of one colony was diluted 1:1000 in 2× YT media (100 µg/ml ampicillin and 12.5 µg/ml tetracycline) and induced after 2 h with 40 µM isopropyl β-d-1-thiogalactopyranoside (IPTG). After a total of 4 h of growth under induction conditions, cells were pelleted by centrifugation at 4000× *g* for 10 min, then washed in one volume of sodium phosphate buffer (PBS) and later re-suspended in 1/100 of the initial volume. A 1 ml aliquot of each induced culture was separated to measure dsRNA concentration. Heat inactivation was performed by heating at 70 °C for 1 h and 200 µl of this suspension was mixed with our ABD (Artificial Bacterial Diet; composition: 40% fish food (Goldfish, Tetra, Germany), 43% guar gum (Sigma-Aldrich, St Luis, MO, USA) and 17% active yeast). Larvae were fed the ABD diet (after L2 stage) for 4 h a day, based on previously published protocols [39]. After this time, the food was removed and cereal (Cerelac 4 Cereals, Nestle) was added as a source of food. Development and survival rates were recorded daily (Additional file 2). All larvae were reared in Petri dishes (25 × 100 mm). In experiments where female pupae and adults were to be counted (4 replicates) or where adult females were to be blood-fed and allowed to oviposit (3 replicates) 20 larvae in each dish for each experimental group were used in each experimental replicate. In each experimental replicate where morphological characters were to be recorded (3 replicates) 30 larvae were used in each dish per experimental group.

To quantify the dsRNA production and to calculate the dsRNA delivered daily by feeding, cell lysis was performed by resuspending the pellet from the cell culture aliquot in 50 µl of 0.1% SDS and incubating at 100 °C for 2 min. To eliminate mRNA, 2 µg of RNase A and 64 µl of RNase A buffer (300 mM NaCl, 10 mM Tris, 5 mM EDTA) were added. After incubation at 37 °C for 5 min, 500 µl of Trizol® (Thermo Fisher Scientific) were added and a protocol of extraction was performed according to the manufacturer’s instructions. The final dsRNA pellet was resuspended in 20 µl of water, incubated at 60 °C for 2 min. One µl was used for spectrophotometric measurement using a Nanodrop (Thermo Fisher Scientific), and 5 µl were used for quantifying on gels using 1.5% agarose.

### Gene expression analysis

RNA was extracted from whole body or dissected tissues using RNeasy Mini Kit (Qiagen, Germantown, MD, USA) according to the manufacturer’s protocol. The complementary DNA was synthesized using the High-Capacity cDNA Reverse transcription kit (Applied Biosystems). The qPCR was performed in a QuantStudio6 Real Time PCR System (Applied Biosystems) using the Power SYBR-green PCR master MIX (Applied Biosystems). The Comparative Ct method [40, 41] was used to compare the changes in the gene expression levels. The *An. gambiae* ribosomal S7 (GenBank: L20837.1) [42], Actin (VectorBase: AGAP000651) and Elongation factor (VectorBase: AGAP005128) [43] genes were used as endogenous controls. The oligonucleotide sequences used in the qPCR assays are presented in Table 1. Three independent biological replicates were conducted, and all PCR reactions were performed in triplicate using extracts of pools of five whole pupae.

### Analysis of morphological characters

Wing lengths, used as indicators of body size, were assessed in adult mosquitoes fed with dsRNA as larvae [44]. The wings were removed from 10-day-old individuals and analyzed using an EVOS fluorescence microscope from AMG (Thermo Fisher Scientific). Lengths were measured using Image J software [45]. Four biological replicates with internal duplicates were combined for statistical analysis.

### Blood-feeding, reproductive and survival assays

Mated *An. gambiae* adult females, 5–10 day-old, were used for fecundity assays. For cross-mating assays of *dsxF* dsRNA reared larvae with untreated individuals, groups of 20 virgin females were maintained in small cages with 20 adult males for 5 days to allow mating. Blood-feeding was performed through Parafilm membranes using sheep blood in water-jacketed artificial feeders maintained at 37 °C. The insects were starved for 4 h prior to the feeding. Unfed mosquitoes were removed from the cages in all the experiments. Females were allowed to oviposit 24 h after blood-feeding. For assays of individual oviposition, each female was placed in a 25 ml tube with 0.5 mm of water and filter paper to collect the eggs. Eggs were counted and then left in water to evaluate hatching and viability rates. After allowing the blood-fed females to oviposite for 72 h, 60 females were dissected in 0.1 M PBS, pH 7.2. Presence or absence of sperm in spermathecae, used as mating indicator, were observed under a stereomicroscope at 400× and 1000× magnifications.

### Statistical analyses

Unpaired Student’s t-tests were applied where comparisons were made between two treatments or conditions, as indicated in the figure legends. Long rank (Mantel-Cox) test was made to analyze survival curves. Two-way ANOVA with Tukey’s multiple comparisons *post-hoc* test was used for analysis of more than two conditions. All statistical analyses were performed using GraphPad 7 Prism Software (Graphpad Software, CA, USA).

## Results

### *Dsx* sequence identification and expression levels

**Fig. 1:**
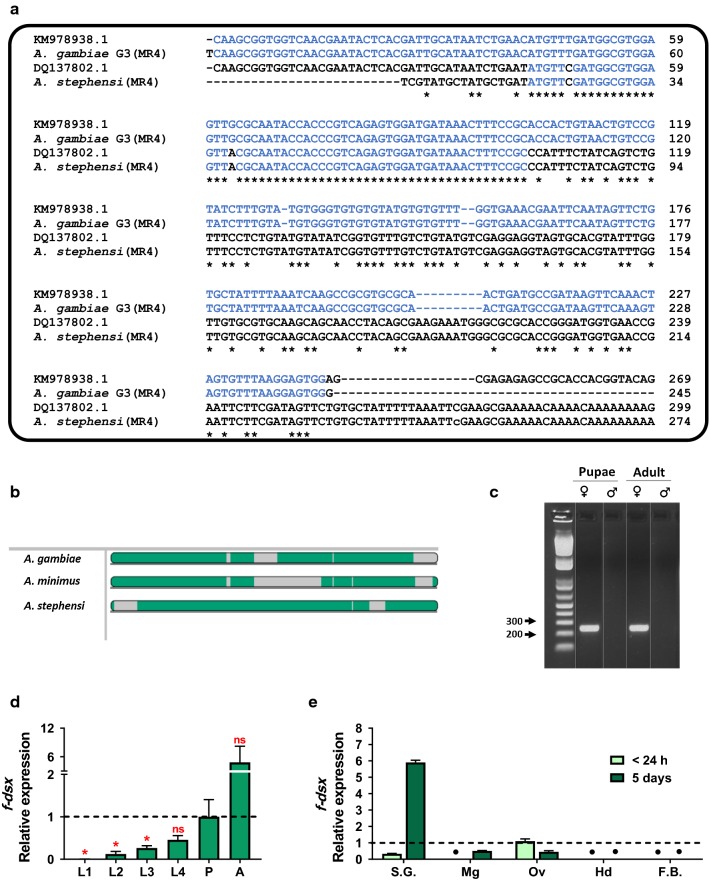
*Doublesex* sequence and profile expression in *An. gambiae* G3. **a** Sequence alignment between the two previously annotated sequences for the *dsx* gene in *An. gambiae* with the sequence of PCR products amplified from the MR4 *An. gambiae* G3 and *An. stephensi* STE2. The *An. gambiae* G3 sequence matched the KM978939 sequence and the *An. stephensi* STE2 sequence matched the DQ137802 sequence. Marked in blue are the sequences that match in at least four consecutive bases the KM978939 sequence for *AgdsxF* exon. Three other regions from our *An. stephensi* strain resulted in 100% match to DQ137802 (Additional file 3). Thus, all subsequent primer designs were based on the KM978939 sequence. **b** Sequence conservation among *Anopheles* for the *AgdsxF* region of interest (1823–2066) was evaluated. PCR products were amplified from *An. gambiae*, *An. minimus*, *An. stephensi* and *An. albimanus* cDNAs (Additional file 3) and the sequences analyzed. A MAFFT alignment is shown, indicating regions with significant similarity in green. **c** PCR amplification of a *AgdsxF* 260 bp fragment, in the fifth exon, showed that the expression was limited to females (using cDNAs of whole body extract) of each sex, consisting of three 1-day-old pupae or five 5-day-old adults. **d** Real-time PCR (RT-PCR) analyses of relative gene expression reveal low levels of *AgdsxF* expression between the L1 and L3 stages, and significantly higher from L4 forward. Two-way ANOVA with Tukey’s multiple comparisons test was performed. **e** Gene expression analysis for *AgdsxF* by qPCR of different tissues. In adult female mosquitoes less than 24 h post-emergence (virgin females), *dsxF* expression was detected only in ovarian tissue (Ov) and salivary glands (SG). In mature adult females (more than 5-days-old and mated) detectable levels of *AgdsxF* expression were observed in the midgut (Mg) and ovaries (Ov), and especially high levels in the salivary glands (SG). Black points indicate the samples that had no detectable expression. *AgdsxF* expression was not detected in either time-point in the head, including antennas and proboscis (Hd) or fat body (FB). Pools of tissue from 12–15 individuals were analyzed

RNAi strategies are based on the delivery of dsRNA complementary to the target gene. We limited our search to previously identified sequences for the *dsx* gene in *D. melanogaster* and other insect species, including *An. gambiae* [18, 24, 25, 27, 28, 46, 47]. A GenBank search (NCBI) for the *An. gambiae* nucleotide sequence of the male and female spliced *Agdsx* variants returned two different results. The original annotated *Agdsx* female specific sequence [24], DQ137802, differs significantly from the one annotated in 2015 (KM978938) by Liu et al. [25]. Liu and collaborators suggested that the former sequence may belong to *An. stephensi*, as genome searches with the former using BLAST matched more closely to this species. To select the sequence for dsRNA production and for qRT-PCR assays, we designed primers (Table 1) on the conserved region at the fifth exon and used them to amplify cDNA obtained from *An. gambiae* G3 (MRA-121, BEI Resources). The resulting sequence is presented in Fig. 1a and was shown to be a match for the KM978938 *AgdsxF* sequence. We used the same primers to amplify cDNA from *An. stephensi* STE2 (MRA-128, BEI Resources), and the sequence was identical to DQ137802. Finally, to determine the nature and variability of the selected region, we tested the same primers with *Anopheles albimanus* STECLA (MRA-126) and *Anopheles minimus* (MRA-729) cDNAs. Due to the phylogenetic distance between these species we were not able to amplify this region from *An. albimanus* but we obtained a 255 bp product from *An. minimus*. The Multiple Alignment using Fast Fourier Transform (MAFFT) of the amplified fragments from the three species differed from one other (Fig. 1b).

The region selected was confirmed to be female-specific (Fig. 1c) and to have the expected expression profile with detectable expression across the different life stages (Fig. 1d), with significantly higher levels of the transcript in adult females when compared to pupae (used as basal condition), and other life-stages (*F*_(5,10)_ = 4.966,* P* =  0.0152). Finally, to determine if the *AgdsxF* is tissue and age-specific in *An. gambiae* G3 adult females, we measured its expression in different individual tissues (salivary glands, midgut, ovaries, fat body, and heads including proboscis and antennae). From mosquitoes at less than 24 h post-emergence and 5 days post-emergence (Fig. 1e) we found that *AgdsxF* expression was tissue-specific and depended on mosquito age, with the most abundant transcript found in the salivary gland of 5-day-old females (*F*_(4,10)_ = 1263, *P* < 0.0001).

### *Dsx* expression profile and silencing through RNAi in larval stages

**Fig. 2:**
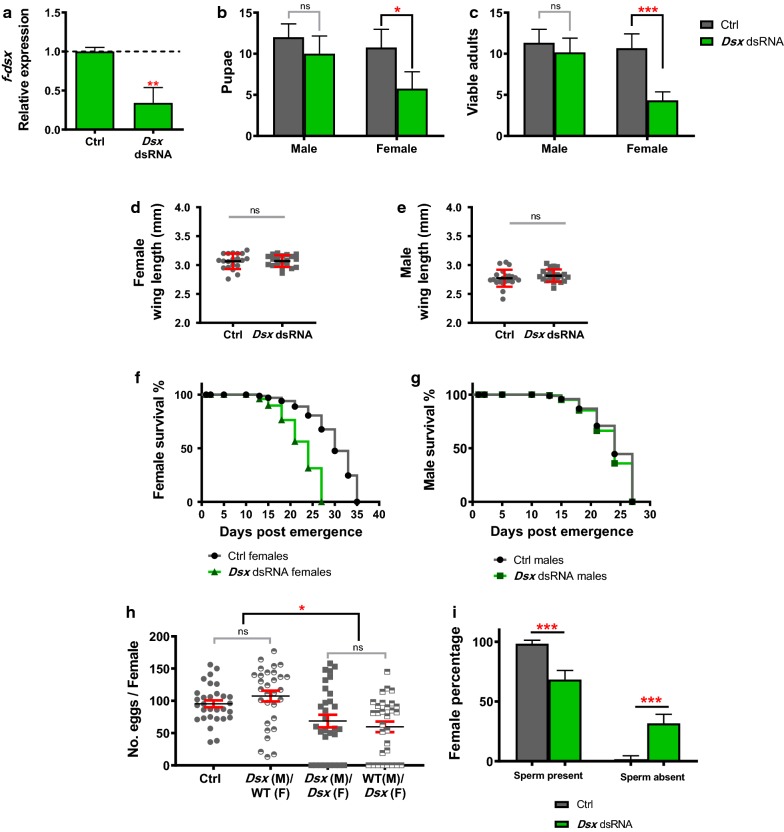
Feeding larvae *dsxF* dsRNA resulted in mRNA inhibition and lower adult female counts with reduced lifespan and fertility. Daily feedings of *AgdsxF* dsRNA to larval stages (from L2 to L4) resulted in a reduction of 66% (± 9.98 SE) of the *AgdsxF* transcript in pupae (**a**). Statistical difference was calculated by unpaired Student’s t-test with *P* < 0.05. **b** The silencing of the *AgdsxF* gene in the larval stages resulted in a reduction of the total number of female pupae (47% ± 3.0% SE of female pupae in the control *vs* 36% ± 5.6 SE in the *dsx* dsRNA-treated group) and subsequently, of the (**c**) adult females (48% ± 4.0% SE of female adults in the control *vs* 27% ± 3.3% SE in the *dsx* dsRNA-treated group). Results are from four independent replicates. **d** Body size, measured by wing length, did not show significant changes in the resulting adult females. **e** Life span of the adult females was significantly reduced by over 5 days. For the males from the treated *AgdsxF* dsRNA groups, no significant variation in body size (**f**) or adult longevity were observed (**g**). The fecundity of the *AgdsxF* dsRNA females (**h**) was significantly lower when compared to that of the control group and that of control females mated with *dsxF* dsRNA males. **i** Sperm were present in the spermathecae of females that laid eggs and absent in those that did not. Of the control females, 2% ± 1.66% SE did not have sperm whereas 30% ± 4.7% SE of *AgdsxF* dsRNA females did not. Phenotypic assays were done in parallel with the qRT-PCR for the treated groups. Except when specified, results are a biological triplicates and in all experiments the control group was fed with a non-related dsRNA, for the *Aintegumenta* gene from *Arabidopsis thaliana*

Because the *AgdsxF* gene is expressed across the different life stages in *An. gambiae*, including larvae, we used oral delivery of *AgdsxF* dsRNA, produced in an *E. coli* HT115 (DE3), a bacterial strain modified for dsRNA production. We fed the larvae with the Artificial Bacterial Diet (ABD), with approximately ≤ 2.3 µg of dsRNA per day, from L2 to L4 stages. A 66% (± 9.9% SE) gene knockdown was confirmed in the pupal stage (Fig. 2a) using the ∆∆Ct method (*AgdsxF* dsRNA M = 0.3417, SD = 0.1969 and Ctrl dsRNA M = 1.001, SD = 0.05223; *t*_(5)_ = 6.592, *P* = 0.0012). As RNA-dependent RNA polymerase mediated transitive amplification is absent in *An. gambiae* [48], we did not expect a long-lasting effect and the phenotypes observed can be considered a result of the RNAi from the larval stages.

### Phenotype description of the *dsxF* silencing by larval feeding

Feeding larvae with *AgdsxF* dsRNA to induce gene silencing resulted in a distortion of the typical ~ 1:1 male to female pupae ratio (Fig. 2b). The percentage of the male and female adults, 52% (± 3.9% SE) and 48% (± 4.0% SE), respectively, in the control (*n* = 80) was significantly different in the *AgdsxF* dsRNA (*t*_(10)_ = 3.303, *P* = 0.03243). Groups fed with *AgdsxF* dsRNA resulted in twice as many males than females reaching adulthood: 72.1% (± 4.0% SE) males *vs* 27.8% (± 3.3% SE) females (*t*_(10)_ = 7.631, *P* = 0.000036) (Fig. 2c). From the mosquitoes that reached adulthood, we evaluated different parameters such as body size, lifespan and fecundity (Fig. 2d-i). The females resulting from *AgdsxF* dsRNA-treated larvae had shorter lifespans (Long-rank test, *χ*^2^ = 127.8, *df* = 1, *P* < 0.0001) and reduced fecundity [male Ctrl dsRNA mated with Ctrl dsRNA females *vs.* male *AgdsxF* dsRNA mated with Ctrl dsRNA females, *t*_(58)_ = 1.203, *P* = 0.2340; male Ctrl dsRNA mated with Ctrl dsRNA females *vs* male *AgdsxF* dsRNA mated with *AgdsxF* dsRNA females, *t*_(58)_ = 2.401, *P* = 0.0196; male Ctrl dsRNA mated with *AgdsxF* dsRNA females *vs* male *AgdsxF* dsRNA mated with *AgdsxF* dsRNA females, *t*_(58)_ = 0.7006, *P* = 0.4863], 37.1% less than the controls, ± 8.2 SE. However, no difference in body-size was found (*t*_(38)_ = 0.1198, *P* = 0.9053).

To confirm that the method was female-specific and did not affect males, the same parameters were recorded for the males (Long-rank test, *χ*^2^ = 1.061, *df* = 1, P = 0.3030 for survival curve and *t*_(40)_ = 1.113, *P* = 0.2725 for wing size) (Fig. 2e-f). Our results not only confirm that the *AgdsxF* gene of *An. gambiae* can be downregulated through RNAi, but also that it is possible to reduce the total number of adult females and to impact their fitness, without affecting male fitness, using RNAi.

## Discussion

The importance of the *dsx* gene has been extensively described in metazoans [20, 49], and many of the key genes involved in the sex determination pathway have been elucidated [21]. In insects, it was first described in *D. melanogaster* by Baker et al. [18] in 1988, and subsequently in many other insect species from the orders Lepidoptera, Coleoptera, Hymenoptera and Diptera [21, 26, 49–51]. Interestingly, until very recently only two references had described and studied the expression of *dsx* sequences for *An. gambiae* [24, 25]. Because of the significant differences among the annotated sequences, we analyzed a conserved region using four different *Anopheles* species (*An. albimanus* STECLA strain, *An. gambiae* G3 strain, *An. minimus* strain and *An. stephensi* STE2 strain (Fig. 1a, b). Our results showed that the sequence product of our cDNA from *An. stephensi* STE2 presented 100% identity to the DQ137802.1 sequence published by Scali et al. in 2005 [24], who had published it as an *An. gambiae* sequence. The sequence we obtained from our *An. gambiae* G3 strain, presented 100% identity to the KM978938.1 sequence annotated by Liu et al. in 2015 [25]. In that work, Liu and collaborators proposed that the DQ137802.1 sequence belonged to *An. stephensi* but did not corroborate the fact experimentally. It is important to note that there are several regions in this female-specific exon that are highly conserved among these diverse *Anopheles* species, but that the sequence variation becomes more significant when larger areas of the sequences are analyzed. Additionally, the *An. stephensi* sequence, as annotated by Scali et al., was successfully used to design a transgenic *An. gambiae* line *dsx-*GFP, where the male mosquitoes selectively expressed high levels of eGFP [6].

To discern the expression pattern of the *AgdsxF* gene in *An. gambiae* G3 strain (Fig. 1b-d), we evaluated male and female individuals, different life stages, and different tissues. As expected, tissues with female-specific functions, i.e. those involved in the blood-meal acquisition and digestion like the salivary glands and the midgut, and the ovarian tissue, presented detectable expression of *AgdsxF*. However, we were not able to detect the transcript either in the head (including the antenna and the mouthparts) or in the fat body. This suggests that *AgdsxF* expression in *An. gambiae* G3 strain is not constitutive, and upstream regulators similar to TRA2 may induce tissue- and time-specific expression according to metabolic and reproductive needs. It is also possible however, that these tissues express the gene at very short and specific time-points, different from the ones that were selected for analysis.

In addition to sequence analysis and sex-specific splicing determination, functional studies of the gene in other mosquito species, such as *Aedes aegypti*, have been done in adult mosquitoes through injection of small interfering RNAs or using soaking and feeding of dsRNA in larvae [28, 33]. As a target for RNAi, the *AgdsxF* gene presents specific challenges for the design of long dsRNA, due to the GC content and the common regions with the male-specific spliced sequence. However, because systemic RNAi in *An. gambiae* has been reported to be successful using long dsRNAs [35, 52–55], we cloned a 260 bp fragment of the female-specific mRNA and selected it for the RNAi template. The use of long dsRNAs may be more stable during the heat-killing procedure of the bacteria. However, the use of short hairpin constructs (shRNA) for dsRNA production, as has been done for other mosquitoes like *Ae. aegypti* [28], could have different results and should be investigated.

There are interesting options of encapsulation to improve delivery of the *AgdsxF* dsRNA, such as chitosan nanoparticles [45] that could potentially improve silencing efficiency. More recently, Kyrou et al. [30] worked with *AgdsxF* to develop a CRISPR-Cas9 line where females were targeted, and performed an analysis of the *dsx* sequence in the female-specific region (exon 5) among members of the *An. gambiae* complex. The conservation of the initial portion of the exon was used as an indicator of the importance of the region on the function of the protein, which allowed for a successful design of a CRISPR-Cas9 gene drive to interfere with female development. The region selected for that construct overlaps with the region selected for RNAi in our work, reinforcing the selection of this sequence as a target for female elimination.

As evidenced by the CRISPR-Cas9 experiments performed in *An. gambiae* [30], the *AgdsxF* gene is haplosufficient. This means that in heterozygous individuals where the *AgdsxF* is interrupted on one chromosome, the females retain normal fecundity and physiology. The implication of this information for RNAi experiments is significant, suggesting that RNAi silencing of less than 50% would not have noticeable phenotypes. Furthermore, the RNAi efficiency depends on the combination of dsRNA size and concentration, point of entry to the organism, and tissue uptake [32]. If tissues with little or no expression of *AgdsxF* take and process the dsRNA available, it is possible that insufficient dsRNA reaches target tissues where more *AgdsxF* mRNA is present. In our experiments, feeding larvae of *An. gambiae* G3 with heat-killed bacteria with *AgdsxF* dsRNA induced a 66% reduction of the *AgdsxF* transcript, resulting in a male-biased sex ratio and reduced lifespan and fecundity in females. It is important to note, that the silencing from each tissue could be different, and the results were obtained from analyzing the whole body of pupae.

The RNA-dependent RNA polymerase mediated transitive amplification that prolongs and spreads RNAi in some insects is absent in *An. gambiae* [48]. Therefore, we expected a short residual effect and we propose that the effects observed are probably due to the RNAi events happening during the larval and pupal stages. Injection of dsRNA into adults, the traditional delivery method of dsRNA to study mosquito biology [52–55], may have different effects but was not applied for this study since the results would not be comparable to the ones obtained by oral delivery in a larval stage. In previous studies with *Rhodnius prolixus*, vector of Chagas disease, we have observed that the RNAi effect for the same gene results in different phenotypes depending on the delivery method and the half-life of the protein that was silenced [38, 56]. Oral delivery of dsRNA using chitosan nanoparticles has been reported in *An. gambiae*. Bacteria producing dsRNA has been used for RNAi in other mosquitoes and insects [29, 32, 33, 35, 38, 39]. Building on previous experience with other insects, we adapted the bacterial delivery of dsRNA as a feasible RNAi methodology in mosquito larvae. Therefore, these methodologies can be adapted to aid in many strategies of vector control for different diseases.

Oral delivery of *AgdsxF* dsRNA to the *An. gambiae* larvae resulted in a reduction of female individuals reaching adulthood. How this reduction in *AgdsxF* transcript interferes with development is poorly understood. In *Ae. aegypti*, *dsxF* upregulates genes involved in the cell cycle in females [57], and in *Cyclommatus metallifer* the *dsx* gene has been linked to the juvenile hormone signaling pathway [58]. It is possible that in *An. gambiae* similar relationships exist, and during the silencing of the *dsx* gene, these pathways or others are being affected. Further work to characterize the cellular processes affected by the gene in *An. gambiae* and other mosquitoes could be of use to design better targets for female elimination.

One of the main findings of this work is the total reduction of the adult females in the treatment group fed with *AgdsxF* dsRNA. This approximately 50% reduction in females is significant, but is unacceptable in an effective SIT program. Risk perception and public assurance that the releases are safe, requires that the female elimination procedures remove females to < 1.0%. The lifespan of females produced in the *AgdsxF* dsRNA treatments is also significantly compromised which could mean that if there were female contaminants, they would likely die sooner in the field. However, the life-span reduction (of ~ 7 days) from the remaining viable females is not enough to indicate that it would necessarily have an impact on parasite transmission.

About 30% of the females that fed on *AgdsxF* dsRNA did not lay any eggs, and after examination of the spermathecae of these individuals we found that the capsule was empty (*t*_(4)_ = 6.364, *P* = 0.003126). The lack of sperm suggested an unsuccessful mating. In *D. melanogaster,* silencing *dsx* expression in neurons makes females unreceptive to courtship or copula [59], and therefore a similar effect could have occurred in this case. More information about the sex differentiation pathway in *An. gambiae* is still needed to understand mating behavior.

Coupling the effect of this specific construct with short hairpin RNAs will be investigated to determine if a stronger effect on phenotypes can be achieved. In summary, feeding larvae with an appropriate target sequence of *AgdsxF* dsRNA is a promising method for removing females from cohorts of mass reared *An. gambiae* sterile males for release. This technology is especially appealing for regions where the continuous exposure to insecticides has made the vectors resistant to traditional control methods. Future studies will focus on improving silencing and dsRNA delivery to facilitate the production of male mosquitoes for release in SIT, gene drive, or other alternative vector control methods.

## Conclusions

Knockdown of *AgdsxF* in *An. gambiae* females after ingestion of *AgdsxF* dsRNA was observed. The total number of adult females was reduced, and their lifespan and fecundity were also negatively affected. Nevertheless, the life-span reduction was not enough to suggest a significant impact on parasite transmission. Fertility was also reduced, but sterility was not achieved. More information about the sex differentiation pathway is still needed, as some parts remain poorly understood. Coupling the effect of this specific construct with different gene targets could potentially result on a stronger effect. This technology for female elimination is particularly useful in new methods in which only males are released. Such methods are promising where the continuous exposure to insecticides has selected for resistance to traditional control methods. Future studies will focus on improving silencing and dsRNA delivery to facilitate the production of male mosquitoes for release in SIT, gene drive, or other alternative vector control methods.

## Additional files

**Additional file 1.** GenBank and VectorBase sequences for *dsx* female splice isoform.

**Additional file 2.** Pupation of male and female groups of *An. gambiae* fed with control dsRNA or *F-dsx* dsRNA.

**Additional file 3.** Comparison of *Doublesex* sequence analysis in four *Anopheles* species (*An. stephensi* STE2, *An. gambiae* G3, *An. minimus* strain and *An. albimanus* STECLA).

## Abbreviations

ABD: artificial bacterial diet
Dmrt: doublesex/mab-3 related transcription factors
dsRNA: double stranded RNA
*dsx*: doublesex gene
*AgdsxF*: female specific *Anopheles gambiae doublesex* gene
FB: fat body
IPTG: Isopropyl β-d-1-thiogalactopyranoside
L1-L4: larvae from stage 1 to 4
MG: midgut
MR4: Malaria Research and Reference Reagent Resource
BEI Resources: Center/Biodefense and Emerging Infections Resources
OV: ovaries
qPCR: quantitative PCR
qRT-PCR: quantitative real-time PCR
RNAi: RNA interference
SG: salivary gland
SE: standard error
SIT: sterile insect technique
