# Supplementary material for: Downregulation of female doublesex expression by oral-mediated RNA interference reduces number and fitness of Anopheles gambiae adult females
Source: Parasit Vectors. 2019 Apr 15;12:170. doi: 10.1186/s13071-019-3437-4 (PMC6466716; doi:10.1186/s13071-019-3437-4)
Supplement: Supplementary file 1 — Additional file 1. GenBank and VectorBase sequences for dsx female splice isoform. [file 13071_2019_3437_MOESM1_ESM.pdf]

<u>Additional File 1</u>

1) *doublesex* sequence information

<u>NCBI GenBank deposited sequence as female *dsx* of *Anopheles gambiae*</u>

>KM978938.1 Anopheles gambiae female-specific doublesex mRNA, complete cds, alternatively spliced

ATCTCCCAAACCCTAGCACCATCGCTTCCTCGACCAACCAATACCGACAGCCCGAGAACGATCGTACCCGAGTGGAAAAC
ACATTGTATTTTCGCAGCAAAAACAACACAGAAATCTTTAAATATTTTAAGATAAACTCCATGTCCCGACAAATCTGCTT
TTTTGCGATTACATAGTAAAGAAACACAGTAGTGAGGAGCTTACTTTTGCTCGTGCTCGTACCACCTTTTAAAAAAACCC
GGAGGGACAATGCCGTCACGCACCACGGCCAACGATTTGCGCGAGCTCGATGTAGCGCCGGCAAGTGTAACGTTAGATCA
AGCTTCCAGATGTTGAGAGTCGGAGTCACAATACGTCCACAACTGTCGGTTCGTCCAATCTGTACATTGTGTGGTCGGTG
TTTGGTGGGAATGACAACGGTGTGTCCTCTTCGAAGGTGCTAAAAGGAAGCTCGCTGACGAGGCGGTAGGGTGTGAGAGT
TTGGCCAGTTTGTTGTTGCGCTTGTGTGGGGTGCAGCAGGGAAAGCATTAGCCGAGAGGTAGAGACACACAAGCTATTTG
GGACCGTGAAATACGCCGCGCGCAACAGTAATAACATAACGTACCGTAAGCCGAAGCGATCGAATCGTGTAATCGAAGCG
GTCTCGTGTTTTTTTCCTCCTATATCGAGAGGCCAACCGATACATCCAGGTGCATTCGGCGGCATAGATAACGCAGCATT
AAGAGTCGGAATTGGCTCTCGAACGCAACAGTTTGATTGATATATAGGCAAGGCGTAGTCAGAGAGGTGCTGTAAACGAG
AAGAAAGTAAGGCTAGCAGGAGAAGCGCAAGTTGAGGAGGGGTGTCGCAGGGTTGACGTAGACGTAGAGCTTGTTTGGAA
GACATACGCGGAACCACACGGGCGTGTGGTGCATCTTGAATGGTGTCACAGGACCGCTGGACGGAAGCAATGTCCGACTC
CGGGTACGATTCGCGCACGGACGGCAACGGTGCGGCCAGCTCGTGCAACAACTCGCTGAACCCGCGGACGCCGCCGAACT
GCGCCCGTTGCCGCAACCACGGGCTGAAGATCGGGCTGAAGGGCCACAAGCGGTACTGCAAGTATCGCGCCTGCCAGTGC
GAGAAGTGCTGCCTGACGGCCGAGCGGCAGCGCGTGATGGCCCTGCAGACGGCGCTGCGGCGCGCCCAGACCCAGGACGA
GCAGCGGGCACTGAACGAGGGCGAGGTACCTCCCGAGCCGGTAGCTAACATTCACATACCAAAGCTATCAGAGCTGAAAG
ACCTGAAGCATAATATGATTCATAATTCTCAGCCGAGATCGTTCGATTGCGACTCCTCCACCGGATCGATGGCGTCCGCA
CCGGGGACCTCCAGCGTGCCACTGACGATACACCGACGGTCGCCGGGCGTACCGCACCACGTTCCCGAGCCGCAGCATAT
GGGAGCTACTCACTCGTGCGTTTCACCAGAACCTGTAAATCTCCTACCTGACGACGAGCTAGTGAAGCGAGCCCAATGGC
TGTTGGAGAAACTCGGCTACCCGTGGGAGATGATGCCCCTGATGTACGTCATACTGAAGAGCGCCGATGGCGATGTACAA
AAAGCACACCAGCGGATCGACGAAGGTCAAGCGGTGGTCAACGAATACTCACGATTGCATAATCTGAACATGTTTGATGG
CGTGGAGTTGCGCAATACCACCCGTCAGAGTGGATGATAAACTTTCCGCACCACTGTAACTGTCCGTATCTTTGTATGTG
GGTGTGTGTATGTGTGTTTGGTGAAACGAATTCAATAGTTCTGTGCTATTTTAAATCAAGCCGCGTGCGCAACTGATGCC
GATAAGTTCAAACTAGTGTTTAAGGAGTGGAGCGAGAGAGCCGCACCACGGTACAGAAGGGCAGCAGAATGGGTCGGCAG
CCTAGCTGCACTGGTGCGGTGCGTCCGGCATCTCGGGGAGAGGGCGGGGAAATTCTAGTGTTAAATCGGAGCAGCAAAAA
CAAAACAGTGGTCGTCCCGTTCAAGAAACGGCCTATACACACACAGAAAACACTGCAGCATGTTTGTACATAGTAGATCC
TAGAGCAGGTGGTCGCTGCTCCTCGAACGCTCTGGACGCACGACTTTGCGCGTACTTGCGTAGCGTTCCGCCGATCGTGG
GTATTCGTACTGCCACAAGCCCGCTTTCTCCCATGCAATCTCTGCAACCAAACCAACAAACAACAACAAAAAACCAATCG
ACACAATGAATCACACCCCTTTTGTATCATCTGTATATTTCTTGTTCTTTGCGTTCTTTTCTATGTGGCCCACGCCCCGG
CGGGTACGTAATTGCGTCGAAAACCCCGAAAACCCCGGCACATACAGTGTACATACGGTTTGAGGACAACTTTGACCTGC
AGCCCTTCTGGGGTTGCCACGTGTAGCTATACTTGTGAGATCGGGCGCCGACGGTGTAAAGCGCGAATGGCCGCCACACA
GTGTGTCCACTCCAACACTACCCCTCTGGAACTACCCCGTCCAGGGATGCACCGGCTCGGCTCATGCCCCTGCAAAACAG
TCCGGGCTCCACTGTAGTAGCTCCGGCGTTGCTCTGAGAGAAGGATGCCCTTCGAAGTGTCGAAAGCGTGCATTGGGCGT
TCAAGTGTATGTGTGTGTGTTAGGTTTAGCGAGAAACAGCAGCAGTTGCGTGTGCTGAAAAGCGAAGGAGTAATAGAGTG
CATAATGAAAATGAAAATGAAAATGAAGCAAAAGTAGAAGGCGGAGGAGAGCAACCTGTGTTCCACTAGTAGCGAATAGT
TTAGTCTAGTTTCGTCACCAATCAACCTTCCAACCATCGTTCAACCAATACCTGAGTCAACATCGTCATCGTTATCGTGC
CACAACTTTATTAAAAATGAACCTTGTCCGCGCCACCGTAGGGTGATCTGAGACGACCTTTCTTACGGGCGCGACCCACA
TGCCATCGTCACCTTCTCCAATCAAAACCAACAGCCTGTACCGATGGTGTGCAATTGTGCGTGCGTGTGTGTTATTAGCA
AAAAAAGAGAAAGAGACGGCGAGAGAGAGATAGATCGAGATCGAGAGTACAAAAGAGCAGTAGAAATGTTCGTTGTTTGT
TTTCCGTAACACAGTTGTTTAGCCAAAATGGGAATTTCCAATAATCCCGGGGGCGGGGAAATGCGGGAATACTGCGTACA
CACATACATCAATCAAAAAGAAAAATCCTTGCGCTACATCACTACCGTTTGCGCGGTGCTGATCTAGAGCAGACCACTTT
CCACGCCACTCTACAATCAATCAATCTGTGCAGAAGGTAAACGACACATTAAGACCTACGAAGCGCTGGTGAAGTCATCG
CTCGATCCGAACAGCGACCGGCTGACGGAAGACGACGACGAGGACGAGAACATCTCGGTGACCCGCACCAACTCCACCAT
TCGGTCGAGGTCCAGCTCGCTGTCGCGGTCCCGGTCCTGCTCGCGCCAGGCCGAAACTCCCCGGGCCGACGATCGGGCCC
TGAACCTTGACACCAAATTCAAACCATCCGCCAGCAGCAGCAGCACCGGCTGCGATCGGGACGACGGTGACTGCGGCGCG
TTCGACGACAGTGCCTCGGTGGTGCGGGGGCACGGGCGGACGGCCCACAGCACCGGTAGCAGGGGCCGCAGCCACTCGAA
ACGGTACCACACCCTCCCGGCCGAGCACATCGGGAGCCACATGGCGGCCGCCCAGAGTCGATCGCCCGCCCCGGACGACG
AGCCGGTGGTGTCGGTGTCCGTGTACGAGAGCCTGGTCGAAGCGGCCAGCAAAAAGACGCGCACCTTCAGCCCGCTCCGG
GGGGAGGCGGAAGATTTGCATACCGCACGGAAAGCATCGCCCCACGACGAGCGGGACGAGCCGACCCCGGCCCAGCCCTA

```
CGAAGCGTACCTGGAGTCGGTGCGGCGGAGTAAAAAGTGCTTCGCGCTCAAGGATAGCGAGGCGCCGGGCGAGGAGCCGA
CGGGCTACGAGAAGGAGAAGGAGCCGCGCATTCCGTACTCGCTGCCGAAGAGCACCTTCGAGCGGCTCGACCTGCTGAAG
AAACCGAACGGGCTTACGTTTCCGATGTACAAGTACAGCGGGATCGAGCCGAACAACTTTGCCCTGCCGCTGCTGCTGCC
CGGGCTGGAGGCGGTCAACCGGACGCTCTACTCGACGCCCTTCCCGGCCCAGCTCCTGCCGTCCAGTCTGTATCCGTCCG
TTAGCAGCGAGTCCACGACAGTGCCCATGTTCCACACGCACTTTCTCGGGTATCAGCCGCCGCTGCAGCTGCCCCACGTC
GAGCACTTCTATCGGAAGGAGCAGCAGCAGCAGCAGCAACAGCAGCAGGGATTGGCCGAACCAAAGGAACCGACGTCGTC
GTCTTCGCCGGGCAGCAACCGGCTTACGCCACCGAAGGGTGCATTTTTCTACGCGAGTGCGGTGGAAAATTCGCTCACCG
CCCAGCAGGCTTCCATTGCTACCATCCATTAGATCCACACTGCGTCCACTCGCTGTTTGCTGCAGCGTACCGCGGACAGT
GCAGTGTACCGCTGTACAAAAAGATCGATTGGAAATTCACATCAATCATCTGCAAGCCCGAAAGATGCTACCTAGTCGTC
CCCAGCTGTTGCTACTGGAGCTTTGCAAGAGATCCAGCTTTCGTTCCTTATCGATGCACAAAAGGCGCACCCGGAAACGA
AACAAAAATCCAACCCACTCGTCAACGGCCCACACGGCGGGTTGCACTGGAGAAACTCCCACCCTCGTAAGTGCTATCTA
AGCGTTAAATTACCTTCGCCCTTTGTGGTAGAACAAACTAGAAGCAAATGAACGTATTAAAAAAAAAAAAAAAA
```

VectorBase sequence matching female specific *dsx* of *Anopheles gambiae* (Exon 5)

```
>AGAP004050-RB AGAP004050-RB-E5 exon:protein_coding
GTCAAGCGGTGGTCAACGAATACTCACGATTGCATAATCTGAACATGTTTGATGGCGTGGAGTTGCGCAATACCACCCGT
CAGAGTGGATGATAAACTTTCCGCACCACTGTAACTGTCCGTATCTTTGTATGTGGGTGTGTGTATGTGTGTTTGGTGAA
ACGAATTCAATAGTTCTGTGCTATTTTAAATCAAGCCGCGTGCGCAACTGATGCCGATAAGTTCAAACTAGTGTTTAAGG
AGTGGAGCGAGAGAGCCGCACCACGGTACAGAAGGGCAGCAGAATGGGTCGGCAGCCTAGCTGCACTGGTGCGGTGCGTC
CGGCGTCTCGGGGGGAGGGCGAGGAAATTCTAGTGTTAAATCGGAGCAGCAAAAACAAAACAGTGGTCGTCCCGTTCAAG
AAACGGCCTGTACACACACACAGAAAACACTGCAGCATGTTTGTACATAGTAGATCCTAGAGCAGGTGGTCGTTGCTCCT
CGAACGCTCTGGACGCACGGCTTCGCGCGTATTTGCGTAGCGTTCCGCCGATCGTGGGTATTCGTACTGCCACAAGCCCG
CTTTCTCCCATGCAATCTCTGCAACCAAACCAACAAACAACAACAAAAAACCAATCGACAAAATGAATCACACCCCTTTT
GTATCATCTGTATATTCTTGTTCTTTGCGTTCTTTTCTATGTGGCCCACGCCCCGGCGGGTACGTAATTGCGTCGAAAAC
CCCGAAAACCCCGGCACATACAGTGTACATACGGTTTGAGGACAACTTTGACCTGCAGCCCTTCTGGGGTTGCCACGTGT
AGCTATACTTGTGAGATCGGGCGCCGACGGTGTAAAGCGCGAATGGCCGCCACACAGTGTGTCCACTCCAACACTACCCC
TCTGGAACTACCCCGTCCAGGGATGCACCGGCTCGGCTCATGCCCCTGCAAAACAGTCCGGGCTCCACTGTAGTAGCTCC
GGCGTTGCTCTGAGAGAAGGATGCCCTTCGAAGTGTCGAAAGCGTGCATTGGGCGTTCAAGTGTGTGTGTGTGTGTTAGG
TTTAGCGAGAAACAGCAGCAGTTGCGTGTGCTGAAAAGCGAAGGAGTAATAGAGTGCATAATGAAAATGAAAATGAAAAT
GAAGCAAAAGTAGAAGGCGGAGGAGAGCAACCTGTGTTCCACTAGTAGCGAATAGTTTAGTCTAGTTTCGTCACCAATCA
ACCTTCCAACCATCGTTCAACCAATACCTGAGTCAACATCGTCATCGTTATCGTGCCACAACTTTATTAAAAATGAACCT
TGTCCGCGCCACCGTAGGGTGATCTAAGGCGACCTTTCTTACGGGCGCGACCCACATGCCATCGTCACCTTCTCCAATCA
AAACCAACAGCCTGTACCGATGGTGTGCAATTGTGCGTGCGTGTGTGTTATTAGCAAAAAAAGAGAAAGAGTCGACGAGA
GAGAGATAGATCGAGATCGAGAGTACAAAAGAGCAGTAGAAATGTTCGTTGTTTGTTTTTCGTAACACAGTTGTTTAGCC
AAAATGGGAATTTCCAATAATCCCGGGGGCGGGGAAATGCGGGAATACTGCGTACACACATACATCAATCAAAAAGAAAA
ATCCTTGCGCTACATCACTACCGTTTGCGCGGTGCTGATCTAGAGCAGACCACTTTCCACTCCACTCTACAATCAATCAA
TCTGTGCAGAAG
```

Alignment of KM978938.1 and AGAP004050 Exon5

```
KM978938.1          --CAAGCGGTGGTCAACGAATACTCACGATTGCATAATCTGAACATGTTTGATGGCGTGG   58
AGAP004050_E5       GTCAAGCGGTGGTCAACGAATACTCACGATTGCATAATCTGAACATGTTTGATGGCGTGG   60
                      **********************************************************

KM978938.1          AGTTGCGCAATACCACCCGTCAGAGTGGATGATAAACTTTCCGCACCACTGTAACTGTCC   118
AGAP004050_E5       AGTTGCGCAATACCACCCGTCAGAGTGGATGATAAACTTTCCGCACCACTGTAACTGTCC   120
                    ************************************************************

KM978938.1          GTATCTTTGTATGTGGGTGTGTGTATGTGTGTTTGGTGAAACGAATTCAATAGTTCTGTG   178
AGAP004050_E5       GTATCTTTGTATGTGGGTGTGTGTATGTGTGTTTGGTGAAACGAATTCAATAGTTCTGTG   180
                    ************************************************************

KM978938.1          CTATTTTAAATCAAGCCGCGTGCGCAACTGATGCCGATAAGTTCAAACTAGTGTTTAAGG   238
AGAP004050_E5       CTATTTTAAATCAAGCCGCGTGCGCAACTGATGCCGATAAGTTCAAACTAGTGTTTAAGG   240
                    ************************************************************

KM978938.1          AGTGGAGCGAGAGAGCCGCACCACGGTACAGAAGGGCAGCAGAATGGGTCGGCAGCCTAG   298
```

```
AGAP004050_E5      AGTGGAGCGAGAGAGCCGCACCACGGTACAGAAGGGCAGCAGAATGGGTCGGCAGCCTAG   300
                   ************************************************************

KM978938.1         CTGCACTGGTGCGGTGCGTCCGGCATCTCGGGGAGAGGGCGGGGAAATTCTAGTGTTAAA   358
AGAP004050_E5      CTGCACTGGTGCGGTGCGTCCGGCGTCTCGGGGGGAGGGCGAGGAAATTCTAGTGTTAAA   360
                   ************************ ******** ******* ******************

KM978938.1         TCGGAGCAGCAAAAACAAAACAGTGGTCGTCCCGTTCAAGAAACGGCCTATACACA--CA   416
AGAP004050_E5      TCGGAGCAGCAAAAACAAAACAGTGGTCGTCCCGTTCAAGAAACGGCCTGTACACACACA   420
                   ************************************************* ******  **

KM978938.1         CAGAAAACACTGCAGCATGTTTGTACATAGTAGATCCTAGAGCAGGTGGTCGCTGCTCCT   476
AGAP004050_E5      CAGAAAACACTGCAGCATGTTTGTACATAGTAGATCCTAGAGCAGGTGGTCGTTGCTCCT   480
                   **************************************************** *******

KM978938.1         CGAACGCTCTGGACGCACGACTTTGCGCGTACTTGCGTAGCGTTCCGCCGATCGTGGGTA   536
AGAP004050_E5      CGAACGCTCTGGACGCACGGCTTCGCGCGTATTTGCGTAGCGTTCCGCCGATCGTGGGTA   540
                   ******************* *** ******* ****************************

KM978938.1         TTCGTACTGCCACAAGCCC-----------------------------------------   555
AGAP004050_E5      TTCGTACTGCCACAAGCCC----------------------------------------    555
                   *******************
```

Primers used for Real Time PCR (yellow) and dsRNA production (cian)

Table 1. Sequences of PCR primers.

Gene	Vector Base ID	Primer name	Primer sequence	Efficiency (%)
Female doublesex	AGAP004050	newDSX-f	AGAGGGCGGGGAAATTCTAGT	111.19
		newDSX-r	GGGCTTGTGGCAGTACGAATA	
Female doublesex	AGAP004050	dsRNA_dsx-f2 (dsx1586)	CAAGCGGTGGTCAACGAATA	
		dsRNA_dsx-r3 (dsx1589)	GCCCACTCCTTAAACACTACTT	
Female doublesex	AGAP004050	T7dsRNA_dsx-f2	TAATACGACTCACTATAGGGCAAGCGGTGGTCAACGAATA	
		T7dsRNA_dsx-r3	TAATACGACTCACTATAGGGGCCCACTCCTTAAACACTACTT	

Confirmation of sequence specificity in the *A. gambiae* genome

Your search is limited to records that include: Anopheles gambiae (taxid:7165) ▷Full Entrez Query

Edit and Resubmit | Save Search Strategies | ▷Formatting options | ▷Download | How to read this page | Blast report description

Job title: Nucleotide Sequence (557 letters)

**RID**	56JNXF0B015 (Expires on 02-02 02:21 am)		
**Query ID**	lcl|Query_214823	**Database Name**	nr
**Description**	None	**Description**	Nucleotide collection (nt)
**Molecule type**	nucleic acid	**Program**	BLASTN 2.8.1+ ▷Citation
**Query Length**	557		

Other reports: ▷Search Summary [Taxonomy reports] [Distance tree of results] [MSA viewer]

Graphic Summary

Distribution of the top 5 Blast Hits on 5 subject sequences

Mouse over to see the title, click to show alignments

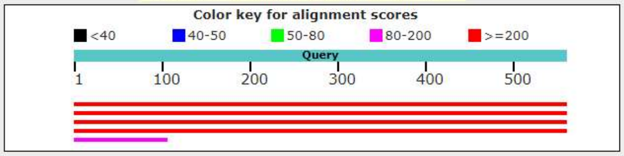

Descriptions

Sequences producing significant alignments:

Select: All None Selected:0

Alignments | Download | GenBank | Graphics | Distance tree of results

Description	Max score	Total score	Query cover	E value	Ident	Accession
Anopheles gambiae str. PEST AGAP004050-RD (AgaP_AGAP004050), mRNA	1029	1029	100%	0.0	100%	XM_003436650.1
Anopheles gambiae str. PEST AGAP004050-RB (AgaP_AGAP004050), mRNA	1029	1029	100%	0.0	100%	XM_309601.5
Anopheles gambiae female-specific doublesex protein (DSX) mRNA, complete cds	977	977	100%	0.0	98%	AY903308.1
Anopheles gambiae female-specific doublesex mRNA, complete cds, alternatively spliced	972	972	100%	0.0	98%	KM978938.1
Anopheles gambiae female-specific doublesex protein mRNA, complete cds	172	172	19%	4e-42	96%	DQ137802.1

Results

Job 275851

CLEAR RESULTS | EXPAND ALL

Description No description available

Submitted Thursday, January 31st, 2019 14:31:57 -0500

Compute Time 3 seconds

Checked Hits

Quick align | Pass to ClustalW | Download

☑ include query

Organism	Database	HSPs
Anopheles gambiae	(Transcripts) PEST strain transcript sequences, AgamP4.10 geneset.	10

Show Query/Hit Numbers

Hit	Gene Name	Description	Query	Aln Length	E-value	Score	Identity	Query Hit	DB Sequence Hit
AGAP004050-RB		Doublesex female isoform		557	0.0	1114	100%		
AGAP007546-RA		4'-phosphopantetheinyl transfera...		32	0.007	44	87.5%		
AGAP008368-RA	TEP14	thioester-containing protein 14		25	0.090	40	92%		
AGAP009016-RA		LIX1-like protein		22	0.32	38	95.5%		
AGAP000046-RA				28	1.1	36	85.7%		
AGAP007502-RA		kinesin family member 11		23	1.1	36	91.3%		
AGAP013082-RA				21	1.1	36	95.2%		
AGAP028433-RA				23	1.1	36	91.3%		
AGAP029050-RA		leucine-rich repeat flightless-inte...		23	1.1	36	91.3%		
AGAP029050-RB		leucine-rich repeat flightless-inte...		23	1.1	36	91.3%		
