# Supplementary material for: Downregulation of female doublesex expression by oral-mediated RNA interference reduces number and fitness of Anopheles gambiae adult females
Source: Parasit Vectors. 2019 Apr 15;12:170. doi: 10.1186/s13071-019-3437-4 (PMC6466716; doi:10.1186/s13071-019-3437-4)
Supplement: Supplementary file 2 — Additional file 2. Pupation of male and female groups of An. gambiae fed with control dsRNA or F-dsx dsRNA. [file 13071_2019_3437_MOESM2_ESM.pdf]

2) Additional File 2

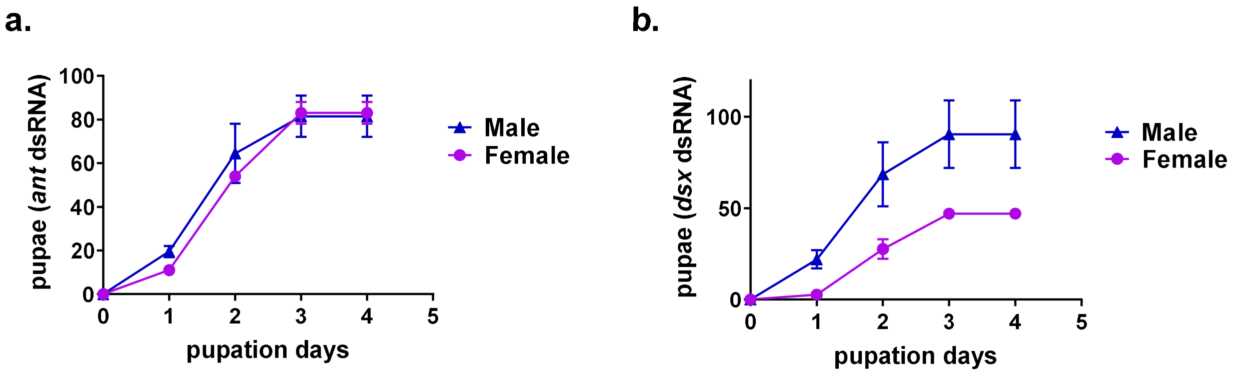

Pupation of male and female groups of *A. gambiae* fed with control dsRNA or *F-dsx* dsRNA. Daily collections of pupae from control groups **(a.)** and *dsx* dsRNA **(b.).** Each day, pupae were collected and sex-determination was done by observation of the pupal terminalia. Results are from four independent replicates, n = 80 (4 x 20).
