# Supplementary material for: Downregulation of female doublesex expression by oral-mediated RNA interference reduces number and fitness of Anopheles gambiae adult females
Source: Parasit Vectors. 2019 Apr 15;12:170. doi: 10.1186/s13071-019-3437-4 (PMC6466716; doi:10.1186/s13071-019-3437-4)
Supplement: Supplementary file 3 — Additional file 3. Comparison of Doublesex sequence analysis in four Anopheles species (An. stephensi STE2, An. gambiae G3, An. minimus strain and An. albimanus STECLA). [file 13071_2019_3437_MOESM3_ESM.pdf]

Additional File 3

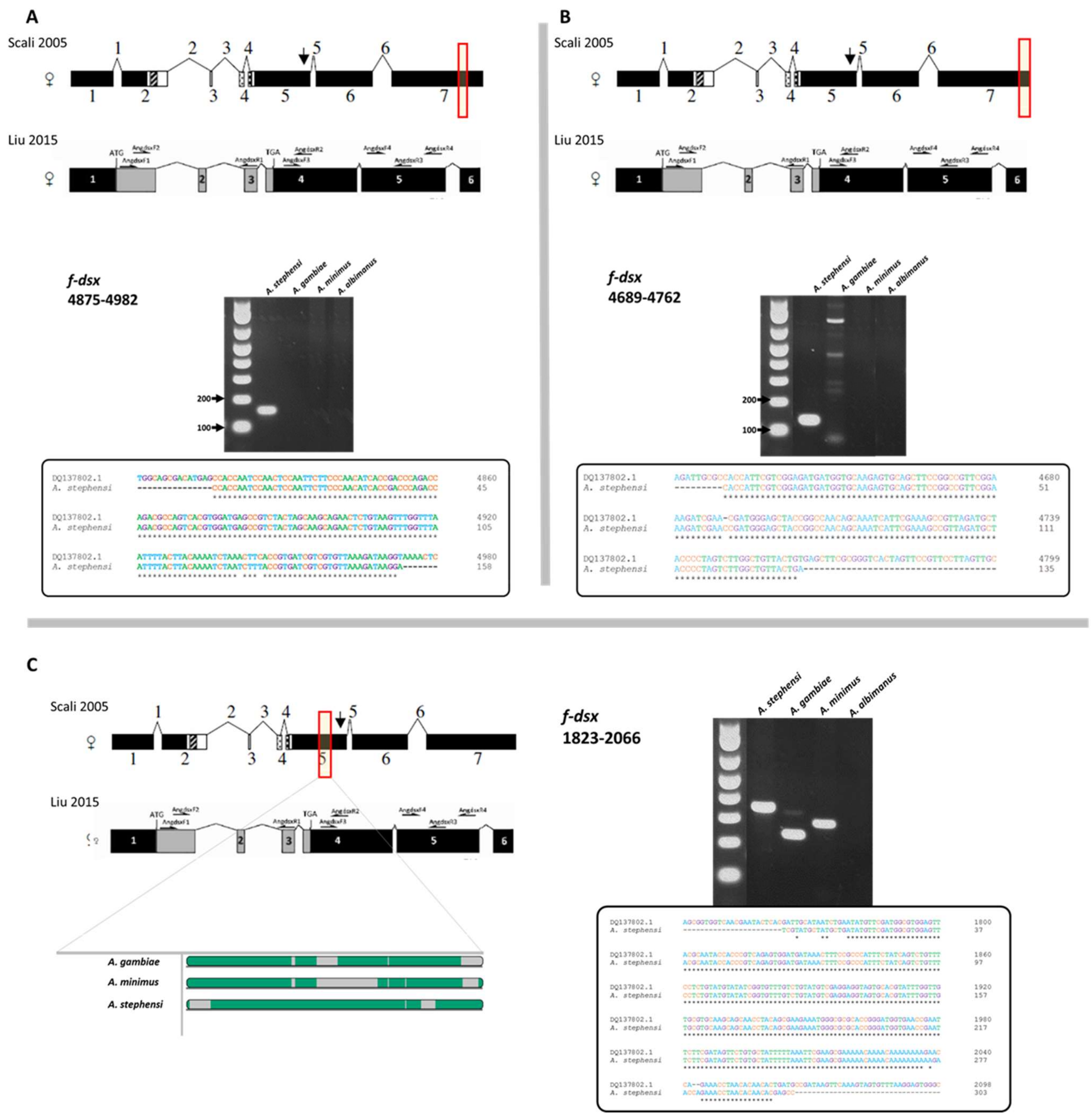

Figure S2. Comparison of *Doublesex* sequence analysis in four *Anopheles* species (*A. stephensi* STE2*, A. gambiae* G3, *A. minimus* strain and *A. albimanus* STECLA*).* Each portion of the figure contains the sequence maps of the gene according to Scali et al. 2005 and to Liu et al. 2015 with a red box indicating the section of the gene target sequence for PCR, the 1.5% agarose gel of the amplified products and the sequence alignment of the that amplified region in *A. stephensi* STE2 and DQ137802 **(a)** Amplification and comparison of the **4689-4762bp region** of the *dsx* gene. **(b)** Amplification of the **4875-4982 bp** region **(c)** Amplification of the **1823-2066 bp region** and the AMAFFT alignment of the whole gene from *A. gambiae*, *A. minimus* and *A. stephensi* with green regions highlighting areas significant similarity
